# Impact of Salinity Stress on Antioxidant Enzyme Activity, Histopathology, and Gene Expression in the Hepatopancreas of the Oriental River Prawn, *Macrobrachium nipponense*

**DOI:** 10.3390/ani15152319

**Published:** 2025-08-07

**Authors:** Shubo Jin, Zhenghao Ye, Hongtuo Fu, Yiwei Xiong, Hui Qiao, Wenyi Zhang, Sufei Jiang

**Affiliations:** 1 Key Laboratory of Freshwater Fisheries and Germplasm Resources Utilization, Ministry of Agriculture and Rural Affairs, Freshwater Fisheries Research Center, Chinese Academy of Fishery Sciences, Wuxi 214081, China; jinsb@ffrc.cn (S.J.);; 2 Wuxi Fisheries College, Nanjing Agricultural University, Wuxi 214081, China

**Keywords:** salinity, oxidative stress, morphological adaptations, immune response, energy metabolism

## Abstract

*Macrobrachium nipponense* represents a commercial decapod species that predominantly inhabits freshwater ecosystems or environments with low salinity. However, the species exhibits normal survival and reproductive capacity in natural aquatic habitats with salinity levels up to 10 parts per thousand (ppt). The present study aimed to elucidate the molecular mechanisms underlying salinity acclimation in *M. nipponense* by investigating alterations in oxidative stress, morphological adaptations, and hepatopancreatic gene expression profiles following exposure to a salinity level of 10 ppt. The present study demonstrates that glutathione peroxidase and Na^+^/K^+^-ATPase play critical roles in mitigating oxidative stress induced by elevated salinity in *M. nipponense*. Furthermore, histological analysis revealed distinct pathological alterations in the hepatopancreas of *M. nipponense* following 7-day salinity exposure, including basement-membrane disruption, luminal expansion, vacuolization, and a marked reduction in storage cells. Transcriptomic profiling of *M. nipponense* hepatopancreas suggested coordinated activation of both immune (lysosome and protein processing in endoplasmic reticulum pathways) and energy (pyruvate metabolism, glycolysis/gluconeogenesis, and citrate cycle) metabolic processes during salinity acclimation in *M. nipponense*. Quantitative real-time PCR validation confirmed the reliability of RNA-seq data. This study provides molecular insights into the salinity adaptation mechanisms in *M. nipponense*, offering potential applications for improving cultivation practices in brackish water environments.

## Simple Summary

The present study elucidates the molecular mechanisms underlying salinity acclimation in *Macrobrachium nipponense* under 10 ppt salinity exposure. Glutathione peroxidase and Na^+^/K^+^-ATPase were identified as key regulators in counteracting salinity-induced oxidative stress. Additionally, histological analysis revealed structural alterations in response to salinity stress, including basement-membrane disruption, luminal expansion, vacuolization, and a significant reduction in storage-cell density. Immune responses and energy metabolism pathways are critically involved in mediating salinity acclimation in *M. nipponense*. These findings provide mechanistic insights into the strategies of *M. nipponense*’s adaptation to osmotic stress at the molecular level.

## 1. Introduction

Saline–alkaline water represents a globally significant but underutilized water resource that is particularly prevalent in inland regions, where it demonstrates low productivity for both aquatic species and agricultural crops. In China alone, saline–alkaline water resources cover approximately 9.91 × 10^7^ hectares, constituting 10% of the global total and ranking third worldwide in terms of distribution area. Distinct from seawater, saline–alkaline water exhibits unique hydrochemical characteristics, including elevated pH levels, high carbonate alkalinity, significant mineralization, complex ionic composition, and limited buffering capacity. These properties render it unsuitable for human and animal consumption, agricultural irrigation, and conventional aquaculture practices [1,2]. Existing research has demonstrated that elevated salinity and alkalinity exert significant physiological stress on aquatic organisms, adversely affecting their survival [3,4]. Consequently, economically valuable aquatic species with limited saline–alkaline tolerance cannot complete their life cycles in these water bodies, resulting in severely constrained aquaculture productivity. Despite China having vast saline–alkaline water resources, current utilization rates remain remarkably low (<2%), representing a significant underutilization of potential aquatic production capacity [5,6]. Given the increasing scarcity of global freshwater resources, there is an urgent need to develop aquaculture technologies for aquatic animals in saline–alkaline water regions. Such advancements would significantly reduce dependence on limited freshwater supplies while ensuring sustainable development of the aquaculture industry. Extensive research has documented the significant impacts of salinity on growth and developmental processes across various crustacean species, including *Mesopodopsis slabberi* [7], *Macrobrachium rosenbergii* [8], *Penaeus monodon* [9], and *Nephrops norvegicus* [10]. These studies have significantly contributed to the development of optimized aquaculture strategies for crustacean species in saline–alkaline aquatic environments.

The oriental river prawn, *Macrobrachium nipponense* (de Haan 1849) (Crustacea: Decapoda: Palaemonidae), represents a commercially significant freshwater prawn species in China, inhabiting freshwater systems and low-salinity estuarine environments nationwide. In 2023, its national production reached 226,392 metric tons, generating substantial economic value. The primary aquaculture regions include Jiangsu, Anhui, Zhejiang, and Jiangxi provinces, each contributing annual yields exceeding 200,000 tons [11]. Palaemonidae crustaceans have undergone multiple independent evolutionary transitions from marine to freshwater habitats [12,13]. Palaemonidae crustaceans maintain stable osmotic homeostasis in freshwater environments through robust cellular regulation mechanisms. The *Macrobrachium* genus exhibit characteristic hyper-osmotic regulation in freshwater/low-salinity environments and hypo-osmotic regulation in high-salinity conditions [14].

Extensive research has been conducted to determine the isosmotic points of various crustacean species. These vary significantly among Penaeidae species, ranging from 21.1 ppt in *Litopenaeus vannamei* [15] to 26.8 ppt in *Litopenaeus setiferus* [16]. Physiological studies have established the salinity tolerance limits of juvenile *M. nipponense* (body length: 2.0–2.5 cm; mean weight: 0.687 g), with median lethal concentrations (*LC*_50_) of 30.71 ppt (24 h), 26.66 ppt (48 h), 26.31 ppt (72 h), and 25.80 ppt (96 h) [17]. Comparative analysis reveals that they have similar 24 h *LC*_50_ values to *Procambarus clarkii* (31.74 ppt) [18], but significantly higher tolerance than *M. rosenbergii* (19.33 ppt) [19], demonstrating notable interspecific variation in osmoregulatory capacity.

The hepatopancreas serves as a primary target organ for assessing environmental stress responses in crustaceans, playing pivotal roles in mediating resistance to diverse stressors including pathogenic bacteria, thermal fluctuations, heavy metal exposure, and osmotic challenges from salinity/alkalinity variations. The hepatopancreas coordinates ion homeostasis, antioxidant defense mechanisms, metabolic reorganization, and detoxification processes to facilitate physiological acclimation to high-salinity aquatic environments [20]. Therefore, it is hypothesized that the dynamic alterations in antioxidant enzyme activity, cellular morphology, and gene expression profiles in the hepatopancreas are critically involved in mediating salinity acclimation in *M. nipponense*. The transcriptome comprises the full complement of RNA transcripts expressed by the genome of a specific cell, tissue, or organism under defined conditions. This approach has been extensively employed to identify candidate genes associated with key traits in *M. nipponense* [21,22,23,24,25]. In this study, we systematically evaluated acute salinity stress effects (10 ppt) on *M. nipponense* hepatopancreas through comprehensive histological examination, quantitative analysis of antioxidant enzyme activities, and transcriptomic profiling analysis after 0, 1, 4, and 7 days of exposure. The present study provides substantive mechanistic insights into osmoregulatory adaptations in *M. nipponense*, offering valuable scientific foundations for developing aquaculture strategies in saline–alkaline regions.

## 2. Materials and Methods

### 2.1. Tissue Collection

A total of 150 healthy prawns with an average body weight of 3.53 ± 0.82 g (mean ± SD) were obtained from the Dapu *M. nipponense* Breeding Base in Wuxi, China (120°13′44″ E, 31°28′22″ N). Prior to the 3-day salinity exposure experiment, the prawns were acclimatized under controlled laboratory conditions, with the water temperature maintained at 26.0 ± 1.2 °C and dissolved oxygen levels kept above 6.0 mg/L. A previous study reported that the maximum salinity tolerance for wild *M. nipponense* was observed in Jingtai, Gansu Province, with a salinity level of 10 ppt [26]. Accordingly, in the present study, the water salinity was adjusted to 10 ppt by dissolving NaCl, while maintaining water temperature at 26.0 ± 1.2 °C, a pH range of 7.29–7.73, and a dissolved oxygen concentration > 6.0 mg/L. Salinity was measured using a calibrated salinity meter. Prawns were maintained in the experimental salinity conditions (10 ppt), and hepatopancreas samples were collected at four time points: baseline (S0, control group; no salinity exposure), 1 day (S1), 4 days (S4), and 7 days (S7) after salinity exposure. For histological analysis, three hepatopancreas samples were collected from each time point and fixed in 4% paraformaldehyde for subsequent sectioning and microscopic examination. For transcriptome profiling, qPCR validation, and antioxidant enzyme activity assays, five hepatopancreas samples were pooled to form one biological replicate, with three independent biological replicates prepared for each analysis. To ensure RNA integrity, all tissue samples were immediately flash-frozen in liquid nitrogen following collection and subsequently stored at −80 °C until further analysis.

### 2.2. Histological Observation

Hematoxylin and eosin (HE) staining was performed to evaluate morphological alterations in the hepatopancreas following 10 ppt salinity exposure. For each time point, three hepatopancreas samples (biological replicates) were processed, with two histological sections (technical replicates) prepared from each sample. The staining protocol followed established methods [27] with modifications as follows: (1) tissue dehydration through an ethanol gradient series, (2) clearing and embedding using xylene–paraffin mixtures, and (3) sectioning at 5 µm thickness using a Leica microtome (Leica Microsystems, Wetzlar, Germany). Sections were stained with HE for 3–8 min and examined using an Olympus SZX16 stereomicroscope (Olympus Corporation, Tokyo, Japan).

### 2.3. Measurement of the Activities of Antioxidant Enzymes

Following acute 10 ppt salinity exposure, antioxidant enzyme activities were quantified using commercial assay kits (Nanjing Jiancheng Bioengineering Institute, Nanjing, China). The measured parameters included total antioxidant capacity (T-AOC, A015-1), superoxide dismutase (SOD, A001-1), catalase (CAT, A007-1-1), glutathione (GSH, A006-1-1), and glutathione peroxidase (GSH-Px, A005-1), along with oxidative stress marker malondialdehyde (MDA, A003-1) and ATPase activities (Na^+^/K^+^-ATPase, A070-2, and Ca^2+^/Mg^2+^-ATPase, A070-3). All assays were performed in accordance with the manufacturer’s protocols using a Bio-Rad iMark microplate reader (Bio-Rad Laboratories, Hercules, CA, USA), and all enzymatic assays were conducted under controlled ambient temperature conditions (37.0 ± 0.5 °C) to ensure experimental consistency.

### 2.4. Transcriptome Profiling Analysis

Transcriptome profiling analysis was conducted to identify salinity-induced gene expression changes in hepatopancreas using the Illumina HiSeq 2500 platform (Illumina, San Diego, CA, USA). Total RNA was extracted from each biological replicate using RNAiso Plus reagent (TaKaRa Bio Inc., Shiga, Japan) according to the manufacturer’s protocol. RNA quality was assessed by spectrophotometric analysis (BioPhotometer D30, Eppendorf AG, Hamburg, Germany) and verified using a 2100 Bioanalyzer (Agilent Technologies, Santa Clara, CA, USA), with all samples demonstrating RNA integrity numbers (RIN) > 7.0. Library preparation utilized 4 μg of total RNA per sample, following established RNA-seq protocols [28,29]. Paired-end sequencing (PE150) was performed via the Illumina HiSeq 2500 platform.

Raw sequencing reads were quality-filtered using fastp (v0.23.2) with default parameters [30]. High-quality reads were then aligned to the *M. nipponense* reference genome (GenBank accession number: GCA_015104395.2) using HISAT2 (v2.2.1) with default mapping parameters [31]. Functional annotation of genes was performed against three databases, Gene Ontology (GO; http://www.geneontology.org, accessed on 9 May 2024) [32], Clusters of Orthologous Groups (COG; http://www.ncbi.nlm.nih.gov/COG, accessed on 9 May 2024) [33], and the Kyoto Encyclopedia of Genes and Genomes (KEGG; http://www.genome.jp/kegg, accessed on 9 May 2024) [34], using a significance threshold of E-value < 10^−5^ [28]. Gene expression levels were quantified as fragments per kilobase of transcript per million mapped reads (FPKM) using HTSeq-count (v0.13.5) [35], calculated as FPKM = (cDNA fragments)/(mapped fragments in millions × transcript length in kb). Differential expression analysis was performed using DESeq2 (v1.38.3) [36], with statistical significance determined by the Benjamini–Hochberg false discovery rate (FDR) correction (q-value < 0.05) [37]. Genes showing ≥2-fold change were classified as upregulated differentially expressed genes (DEGs), while those showing ≤0.5-fold change were considered downregulated DEGs.

### 2.5. qPCR Analysis

RNA-seq data validation was performed using quantitative real-time PCR (qPCR) following established protocols [38,39]. Total RNA was extracted from hepatopancreas samples at each time point using the UNlQ-10 Column Trizol Total RNA Isolation Kit (Sangon Biotech, Shanghai, China). RNA concentration was determined spectrophotometrically (BioPhotometer D30, Eppendorf AG, Hamburg, Germany), and integrity was verified by 1.2% agarose gel electrophoresis.

For cDNA synthesis, 1 μg of total RNA was reverse-transcribed using the PrimeScript™ RT reagent kit (Takara Bio Inc., Shiga, Japan) according to the manufacturer’s protocol. qPCRs were performed using UltraSYBR Mixture (CWBIO, Beijing, China) on a Bio-Rad iCycler iQ5 Real-Time PCR System (Bio-Rad Laboratories, Hercules, CA, USA). All primer sequences are provided in Table 1. The eukaryotic translation initiation factor 5A (*EIF*) gene served as the reference control, having demonstrated stable expression across experimental conditions in *M. nipponense* [40]. Relative gene expression levels were calculated using the 2^−ΔΔCT^ method [41].

### 2.6. Statistical Analysis

All statistical analyses were performed using SPSS Statistics 23.0 (IBM Corp., Armonk, NY, USA). Gene expression levels and antioxidant enzyme activities were compared by one-way analysis of variance (ANOVA) [38,39], with statistical significance set at *p* < 0.05. Prior to conducting ANOVA, the homogeneity of variances was confirmed (Levene’s test, *p* > 0.05). Linear regression analysis was subsequently performed for each dataset. The results demonstrated satisfactory model fit, with residual deviations approximating unity and mean residuals approaching zero across all groups. These diagnostic measures confirm that the residual distributions met the assumptions of normality required for parametric analysis. Quantitative data are presented as mean ± standard deviation (SD).

## 3. Results

### 3.1. Changes in Antioxidant Enzymes After Salinity Exposure

**Figure 1:**
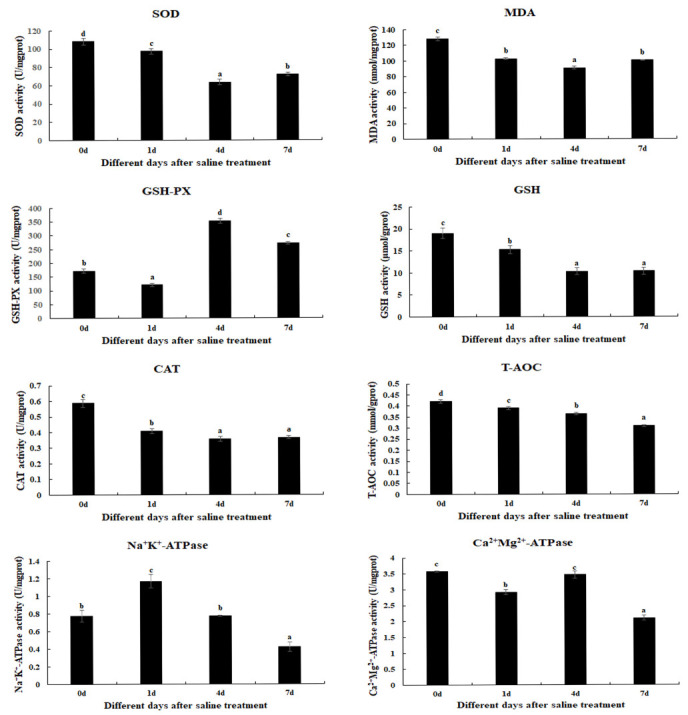
Temporal variations in hepatopancreatic antioxidant enzyme activities in *M. nipponense* following 10 ppt salinity exposure. Data represent mean ± SD (*n* = three biological replicates per time point). Different lowercase letters denote statistically significant differences (*p* < 0.05) among exposure durations. CAT: catalase; GSH: glutathione; GSH-PX: glutathione peroxidase; MDA: malondialdehyde; SOD: superoxide dismutase; T-AOC: total antioxidant capacity.

Antioxidant enzyme activities were quantitatively assessed at 24 h (Day 1), 96 h (Day 4), and 168 h (Day 7) after 10 ppt salinity exposure (Figure 1). Quantitative analysis demonstrated that salinity exposure significantly suppressed (*p* < 0.05) the activities of key antioxidant markers, including SOD, MDA, GSH, CAT, T-AOC, and Ca^2+^/Mg^2+^-ATPase. Notably, GSH, CAT, and T-AOC exhibited progressive, time-dependent decreases in activity that correlated significantly with exposure duration. In contrast, salinity exposure significantly upregulated (*p* < 0.05) both GSH-PX activity, peaking at 96 h after exposure, and Na^+^/K^+^-ATPase activity, reaching maximal levels at 24 h.

### 3.2. Morphological Changes in Hepatopancreas After Salinity Exposure

**Figure 2:**
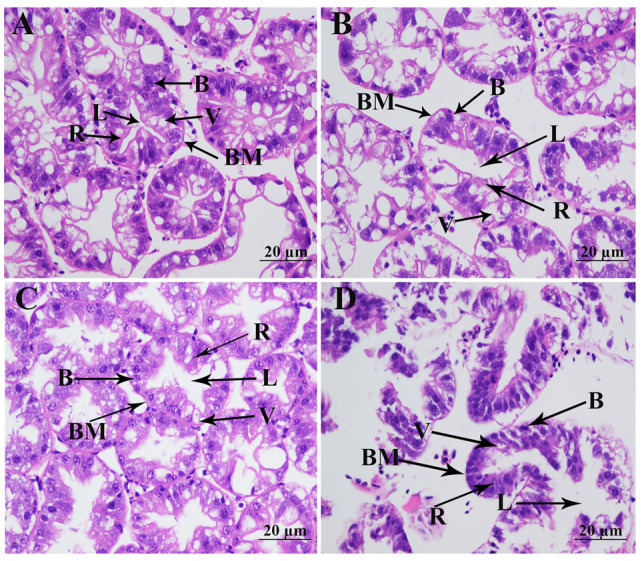
Morphological changes in hepatopancreas under 10 ppt salinity. (B): type B secretory cells; BM: basement membrane; L: lumen; R: type R storage cells; V: transferred vacuoles. Scale bars = 20 µm. (**A**): morphology of hepatopancreas without salinity exposure; (**B**): morphology of hepatopancreas after 1 day of salinity exposure; (**C**): morphology of hepatopancreas after 4 days of salinity exposure; (**D**): morphology of hepatopancreas after 7 days of salinity exposure.

Histopathological analysis via hematoxylin–eosin (H&E) staining revealed significant salinity-induced morphological alterations in the hepatopancreas (Figure 2). The hepatopancreas of *M. nipponense* exhibits a characteristic histological organization, consisting of secretory cells, basement membrane, well-defined luminal spaces, storage cells, and vacuoles. Histopathological examination revealed preserved hepatopancreas architecture following 24 h (Day 1) and 96 h (Day 4) of salinity exposure. However, histopathological analysis demonstrated significant structural alterations following prolonged (168 h) salinity exposure, characterized by severe basement-membrane disruption, marked luminal expansion, pronounced cytoplasmic vacuolization, and a significant reduction in storage-cell density.

### 3.3. Transcriptome Profiling Analysis of Hepatopancreas

In this study, DEGs were identified using stringent fold-change thresholds of >log (>1.0-fold change) as upregulated DEGs and <log (<−1.0-fold change) as downregulated DEGs. Comparative transcriptomic analysis revealed distinct differential gene expression patterns across salinity treatments, including 25 DEGs (3 upregulated, 22 downregulated) between S0 and S1, 301 DEGs (275 upregulated, 26 downregulated) between S1 and S4, and 489 DEGs (367 upregulated, 122 downregulated) between S4 and S7.

**Figure 3:**
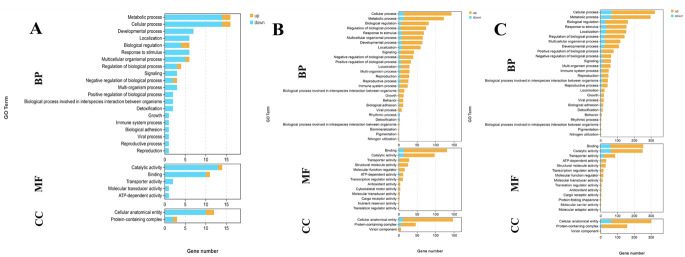
GO analysis of DEGs in the hepatopancreas after different days of salinity exposure under the concentration of 10 ppt. (**A**): GO analysis between S0 and S1. (**B**): GO analysis between S1 and S4. (**C**): GO analysis between S4 and S7. BP: biological process; MF: molecular function; CC: cellular component.

GO analysis identified functional annotations for 19 DEGs in the S0 vs. S1 comparison, 204 DEGs in the S1 vs. S4 comparison, and 318 DEGs in the S4 vs. S7 comparison. GO term enrichment analysis revealed 27 enriched GO terms in the S0 vs. S1 comparison, 43 enriched GO terms in the S1 vs. S4 comparison, and 44 enriched GO terms in the S4 vs. S7 comparison. Five core GO categories were consistently enriched across all comparisons, including cellular process, metabolic process, molecular binding, catalytic activity, and cellular anatomical entity (Figure 3).

**Figure 4:**
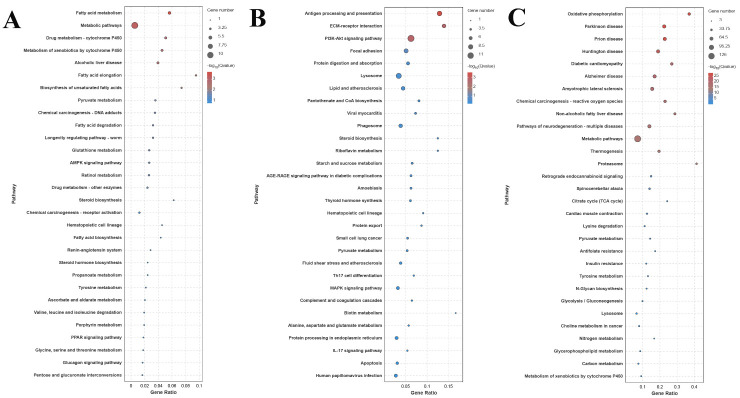
KEGG analysis of DEGs in the hepatopancreas after a different number of days of salinity exposure under a 10 ppt concentration. (**A**): KEGG analysis between S0 and S1. (**B**): KEGG analysis between S1 and S4. (**C**): KEGG analysis between S4 and S7.

KEGG annotation analysis identified functional classifications for 11 DEGs in the S0 vs. S1 comparison, 86 DEGs in the S1 vs. S4 comparison, and 226 DEGs in the S4 vs. S7 comparison. Metabolic pathway enrichment analysis revealed 43 enriched pathways in the S0 vs. S1 comparison, 154 enriched pathways in the S1 vs. S4 comparison, and 201 enriched pathways in the S4 vs. S7 comparison. Comparative pathway analysis identified significantly enriched core metabolic pathways among DEGs in both the S1 vs. S4 and S4 vs. S7 comparisons, including lysosome, protein processing in endoplasmic reticulum, pyruvate metabolism, glycolysis/gluconeogenesis, and citrate cycle (TCA cycle) (Figure 4).

### 3.4. Identification of Candidate Genes Involved in Salinity Acclimation

Candidate genes implicated in salinity acclimation were identified through significant association with the enriched metabolic pathways described above, and differential expression was observed in at least two comparative groups (Table 2). Transcriptomic analysis identified five lysosomal-pathway-associated genes exhibiting salinity-responsive expression patterns, including cathepsin B, cathepsin L, alpha-L-fucosidase (*FUCA*), legumain, and solute carrier family 17 member 5 (*SLC17A5*). No significant differential expressions of these genes were observed between S0 and S1, while progressive upregulation occurred upon exposure from Day 1 to Day 4. Transcriptomic analysis identified two key regulatory components from the endoplasmic reticulum protein-processing pathway exhibiting divergent responses to salinity exposure. Salinity exposure elicited significant transcriptional activation of crystallin alpha B (*CRYAB*) and marked transcriptional suppression of *HSP90A*. S-glutathione dehydrogenase, a key enzyme enriched in both the pyruvate metabolism and glycolysis/gluconeogenesis pathways, exhibited significant downregulation following salinity exposure. Phosphoenolpyruvate carboxykinase (*PEPCK*) was significantly enriched in pyruvate metabolism, glycolysis/gluconeogenesis, and the TCA cycle. Pyruvate dehydrogenase E1 component (*PDHE1*) showed significant enrichment in glycolysis/gluconeogenesis and the TCA cycle.

### 3.5. qPCR Verification of DEGs

**Figure 5:**
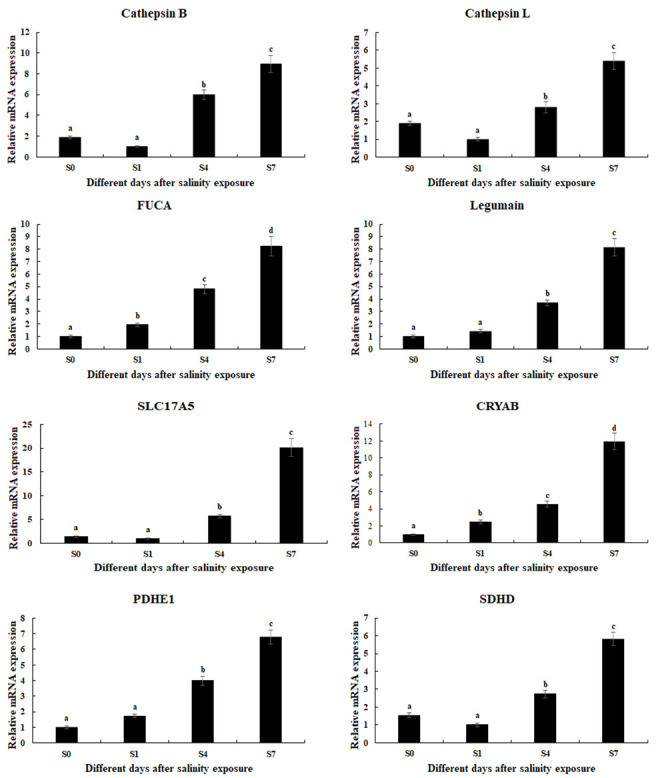
Verification of DEGs expression in the hepatopancreas after different days under 10 ppt salinity treatment by qPCR analyses. Data are shown as mean ± SD (standard deviation) of tissues from three biological replicates. Letters indicate significant differences between different days.

To validate the RNA-seq data, qPCR was performed on eight candidate DEGs. The consistency between qPCR and RNA-seq data confirmed the reliability of the transcriptomic findings (Figure 5). Salinity exposure significantly upregulated the expression of eight key DEGs (*p* < 0.05), exhibiting time-dependent induction patterns that reached maximal levels at 168 h (Day 7) after exposure, including cathepsin B, cathepsin L, *FUCA*, legumain, *CRYAB*, *SLC17A5*, *PDHE1*, and succinate dehydrogenase.

## 4. Discussion

Environmental stressors can trigger oxidative stress responses in aquatic organisms [42,43], resulting in elevated production of reactive oxygen species (ROS). Excessive ROS accumulation induces cellular structural damage, which may manifest as observable histopathological alterations in hepatopancreas tissue [44]. To mitigate oxidative damage, aquatic species have evolved an integrated antioxidant defense system comprising SOD, CAT, and GSH-Px, which collectively function to scavenge ROS and maintain cellular homeostasis under environmental stress conditions [45,46]. Numerous studies have documented significant alterations in antioxidant enzyme activities in aquatic organisms exposed to high-salinity environments [47,48,49,50]. However, the composition and responsiveness of antioxidant defense systems exhibit substantial interspecific variation among aquatic species. The present study demonstrates that 10 ppt salinity exposure significantly upregulates both GSH-Px and Na^+^/K^+^-ATPase activities in *M. nipponense* hepatopancreas (*p* < 0.05). This coordinated induction suggests these enzymes may constitute a crucial biochemical defense mechanism against salinity-induced oxidative stress in prawns. GSH-Px catalyzes the oxidation of reduced glutathione to glutathione disulfide, while simultaneously reducing hydrogen peroxide to water or lipid hydroperoxides to their corresponding alcohols. This coupled redox reaction eliminates reactive oxygen species and protects cellular membranes from oxidative damage by maintaining redox homeostasis [51]. Na^+^/K^+^-ATPase is an ATP-dependent transmembrane transporter that actively exchanges three intracellular Na^+^ ions for two extracellular K^+^ ions per ATP hydrolytic activity. This primary active transport mechanism drives several critical physiological processes, including osmolyte transportation, acid–base homeostasis, nitrogen excretion, and respiratory gas exchange in branchial epithelia. In aquatic organisms, this enzyme mediates critical ion exchange between body fluids and the external environment, thereby maintaining osmotic balance across both vertebrate and invertebrate species [52,53].

Previous studies have demonstrated that salinity exposure induces distinct hepatopancreatic morphological alterations in aquatic species, which may facilitate either physiological adaptation through structural reorganization or pathological degeneration culminating in mortality. As demonstrated in *L. vannamei* [54], elevated salinity induces marked histopathological alterations in the hepatopancreas, characterized by tubular epithelial degeneration and progressive cytoplasmic vacuolization. Histopathological analysis of *Scylla paramamosain* hepatopancreas revealed salinity-dependent cellular alterations [55], including cellular edema, cytoplasmic vacuolization, and necrosis. These pathological manifestations exhibited significant positive correlation with exposure duration, demonstrating the time-dependent exacerbation of tissue damage. Histopathological analysis demonstrated that salinity exposure induced significant inflammation and lipid accumulation in the hepatopancreas of *M. rosenbergii* [56]. In the present study, salinity exposure induced significant structural alterations in the *M. nipponense* hepatopancreas, including basement-membrane disruption, luminal expansion, cytoplasmic vacuolization, and a marked reduction in storage-cell density.

Transcriptome profiling has emerged as a powerful tool for elucidating the molecular mechanisms underlying salinity stress tolerance in aquatic species. Comparative analyses have been conducted across diverse taxa, including *Brassica rapa* [57], red tilapia (*Oreochromis* spp.) [58], *Oreochromis aureus* [59], and *Exopalaemon carinicauda* [60], revealing conserved and species-specific regulatory pathways involved in osmoregulation and ionic homeostasis. Transcriptomic analysis was conducted to investigate the molecular mechanisms underlying salinity acclimation in *M. nipponense*, with a focus on the gills and hepatopancreas under varying salinity conditions. This approach identified key metabolic pathways and DEGs associated with salinity acclimation [61]. In this study, comparative transcriptomic analysis between the S0 and S1 groups identified only 25 DEGs in the hepatopancreas of *M. nipponense*. This limited transcriptional response suggests that acute exposure (24 h) to 10 ppt salinity does not induce substantial gene expression changes in this organ system. Comparative analysis of the S1 vs. S4 and S4 vs. S7 groups revealed a significant enrichment of DEGs in lysosome, protein processing in the endoplasmic reticulum, pyruvate metabolism, glycolysis/gluconeogenesis, and the citrate cycle (TCA cycle). The findings demonstrate that these metabolic pathways exhibit significant responsiveness to salinity acclimation in *M. nipponense*, as evidenced by the progressive alterations in the expression of pathway-enriched genes.

The immune system serves as a crucial regulatory mechanism in salinity acclimation, orchestrating physiological adaptations and preserving systemic homeostasis under osmotic stress conditions [62,63]. The transcriptomic analysis reveals two pivotal immune-related metabolic pathways (lysosome and protein processing in endoplasmic reticulum) that appear to facilitate salinity adaptation in *M. nipponense*. As membrane-bound organelles containing hydrolytic enzymes, lysosomes play a fundamental role in cellular waste processing and turnover [64,65]. Notably, the transcriptomic data demonstrate significant upregulation of key lysosomal proteases, including cathepsin B, cathepsin L, and legumain in *M. nipponense* under salinity stress. These proteases may contribute to salinity adaptation through the modulation of inflammatory responses [66,67,68], clearance of damaged cellular components via autophagy [69], and maintenance of tissue integrity and function [70]. The endoplasmic reticulum (ER) in aquatic species is highly sensitive to environmental stress. When protein folding is disrupted, misfolded proteins accumulate, triggering an unfolded protein response, which is a conserved mechanism that restores ER function [71,72,73]. Transcriptomic analysis revealed two functionally significant genes associated with ER protein processing in *M. nipponense*. *CRYAB*, a small heat shock protein family member that maintains proteostasis by preventing stress-induced protein aggregation [74,75,76,77]. *HSP90*, an ATP-dependent molecular chaperone, facilitates proper protein folding and targets misfolded proteins for proteasomal degradation [78,79,80]. Thus, the DEGs associated with these pathways likely mediate adaptive responses by maintaining cellular homeostasis through proper protein folding and degradation, and supporting essential cellular processes including intracellular digestion and cell cycle regulation, thereby enhancing the organism’s osmoregulatory capacity during salinity acclimation in *M. nipponense*.

Energy metabolism serves as the biochemical foundation for osmoregulatory adaptation during salinity acclimation in aquatic species, providing the necessary ATP to drive active ion transport and cellular homeostasis maintenance [81,82,83]. The transcriptomic analysis reveals three pivotal energy metabolism-related metabolic pathways (glycolysis/gluconeogenesis, pyruvate metabolism, and the TCA cycle) that appear to facilitate salinity adaptation in *M. nipponense*. Glycolysis, an evolutionarily conserved anaerobic pathway present in both eukaryotic and prokaryotic organisms, catalyzes the conversion of glucose to pyruvate with the concomitant production of two ATP molecules and NADH, serving as a critical energy-producing mechanism during environmental stress [84,85]. As its reverse pathway, gluconeogenesis mediates glucose synthesis from non-carbohydrate substrates (including lactate, pyruvate, and glucogenic amino acids) that are primarily in hepatic and renal tissues, thereby maintaining systemic glucose homeostasis during nutrient deprivation [86]. As the terminal product of glycolysis, pyruvate serves as a critical metabolic node that is determined by cellular oxygen availability. Pyruvate converts to lactate under anaerobic conditions, maintaining ATP production. Conversely, pyruvate converts to acetyl-CoA under aerobic conditions, serving as fuel for mitochondrial ATP generation via the TCA cycle. This metabolic flexibility allows cells to adapt to varying energy demands and oxygen availability [87]. The TCA cycle functions as the principal bioenergetic hub in aerobic organisms, generating ATP through oxidative phosphorylation [88], modulating immune responses through metabolic intermediates [89,90], and supplying critical biosynthetic precursors (acetyl-CoA, pyruvate, oxaloacetate (OAA), succinate, and α-ketoglutarate) for cellular metabolism [91,92,93].

Glycolysis/gluconeogenesis, pyruvate metabolism, and the TCA cycle are interconnected pathways governing cellular energy metabolism. The DEGs associated with these pathways suggests their involvement in salinity acclimation by modulating energy provision in *M. nipponense*. Notably, transcriptomic analysis revealed several candidate genes in response to salinity exposure. As s-lactoylglutathione dehydrogenase plays an essential role in maintaining cellular reduced glutathione homeostasis through NADPH-dependent reduction [94], its decreased expression may compromise the organism’s capacity for oxidative stress defense, xenobiotic detoxification, and maintenance of cellular redox balance. *PEPCK* regulates glucose homeostasis through its dual roles in gluconeogenesis and the TCA cycle [95]. It catalyzes the ATP-dependent carboxylation of phosphoenolpyruvate to OAA for gluconeogenesis and CO_2_ fixation [96], and the GTP-dependent decarboxylation of OAA to phosphoenolpyruvate [97], demonstrating remarkable metabolic flexibility in energy metabolism. *PDHE1*, a key component of the pyruvate dehydrogenase complex (PDH), regulates glycolytic pyruvate decarboxylation to mitochondrial energy production [98,99]. Interestingly, PDHE1 also appears to regulate male reproduction in *M. nipponense* [39], suggesting broader physiological roles beyond energy metabolism. The expression patterns of these DEGs were validated via qPCR, corroborating the RNA-seq data and reinforcing the reliability of our findings.

## 5. Conclusions

This study elucidates the molecular pathways and genes underlying hepatopancreatic responses to salinity acclimation in *M. nipponense*. Exposure to 10 ppt salinity significantly enhances the enzymatic activities of GSH-PX and Na^+^/K^+^-ATPase. Furthermore, salinity stress induces distinct structural modifications in the hepatopancreas, characterized by luminal dilation, cytoplasmic vacuolization, and a marked decrease in cell storage density. Transcriptomic analysis identifies key metabolic pathways enriched among DEGs, including lysosome, protein processing in endoplasmic reticulum, pyruvate metabolism, glycolysis/gluconeogenesis, and the TCA cycle. These findings suggest that immune response modulation and energy metabolic reprogramming play pivotal roles in salinity adaptation. Future research will focus on the functional characterization of candidate DEGs to improve the salinity tolerance and aquaculture productivity (growth and reproduction) of *M. nipponense* in hypersaline environments.

## Figures and Tables

**Table 1:** The primers used in the present study.

Gene	Primer
Cathepsin B	F: ATTCCCGAATGCGAGCATCA
	R: CCTCAACGGGGCCATTAGTC
Cathepsin L	F: GCCGGTTTCTGTTGCTATCG
	R: CCATGACTTGCTCCACGAGT
alpha-L-fucosidase	F: CCATTGTTCTCCAGTGGCCT
	R: GTTAATTCCAGCACCCACGC
Legumain	F: TCACTGAACCCAAACCCAGG
	R: CCCAATTCCTTCCATGGCCT
Solute larrier camily 17, member 5	F: GCTTGGCGGTTCGTTTTCTT
	R: AGCTTTTGGCATGAGGACCA
Crystallin, alpha B	F: CGAGTTGCAAGTTCGCGTAG
	R: GCCTTCCCTCTTTGGAGCAT
Pyruvate dehydrogenase E1	F: AATGGGGGCATTTGTGTTGC
	R: AGATGCAGATGCACGGTCAA
Succinate dehydrogenase	F: ACGTGCGCTAATACCTTGTCA
	R: TACTCGATAGCCGGAGACGG
Eukaryotic translation initiation factor 5A	F: CATGGATGTACCTGTGGTGAAAC
	R: CTGTCAGCAGAAGGTCCTCATTA

**Table 2:** Identification of the candidate genes involved in the salinity acclimation of *M. nipponense*.

Gene	Accession Number	Metabolic Pathway	Log (Fold Change)		
			S0 vs. S1	S1 vs. S4	S4 vs. S7
Cathepsin B	MSTRG.22966	Lysosome		2.4	1.8
Cathepsin L	ncbi_135220875	Lysosome		1.1	1.6
alpha-L-fucosidase	ncbi_135221329	Lysosome		1.5	1.3
Legumain	ncbi_135217338	Lysosome		1.4	1.5
Solute larrier camily 17, member 5	ncbi_135224400	Lysosome		2.6	5.1
Crystallin, alpha B	ncbi_135198784	Protein processing in endoplasmic reticulum		1.9	2.4
Heat shock protein 90A	ncbi_135227160	Protein processing in endoplasmic reticulum		−1.6	−1.3
S-lutathione dehydrogenase	ncbi_135202288	Pyruvate metabolism, Glycolysis/Gluconeogenesis	−1.4	−2.1	−1.6
Phosphoenolpyruvate carboxykinase	ncbi_135220828	Pyruvate metabolism, Glycolysis/Gluconeogenesis, Citrate cycle		1.7	−1.3
Lactoylglutathione lyase	MSTRG.6143	Pyruvate metabolism		−2.7	−2.5
Pyruvate dehydrogenase E1	ncbi_135195838	Glycolysis/Gluconeogenesis, Citrate cycle		1.2	1.1
Succinate dehydrogenase	ncbi_135196176	Citrate cycle		2.1	1.3

## Data Availability

The raw data of the present study have been submitted to NCBI with the accession numbers SRX28126544-SRX28126555. All other data are contained within the main manuscript.
